# A radiological study of bone remodeling with two different types of porous β-tricalcium phosphate in humans

**DOI:** 10.1038/s41598-020-77011-3

**Published:** 2020-11-16

**Authors:** Naoya Kikuchi, Tomokazu Yoshioka, Norihito Arai, Kojiro Hyodo, Akihiro Kanamori, Masashi Yamazaki

**Affiliations:** 1 Department of Orthopedic Surgery, Faculty of Medicine, University of Tsukuba, 1-1-1 Tennodai, Tsukuba, Ibaraki 305-8575 Japan; 2 Division of Regenerative Medicine for Musculoskeletal System, Faculty of Medicine, University of Tsukuba, 1-1-1 Tennodai, Tsukuba, Ibaraki 305-8575 Japan

**Keywords:** Medical research, Materials science

## Abstract

In this study we compared the bone remodeling of unidirectional (UDPTCP) and spherical porous β-tricalcium phosphate (SPTCP) radiologically in humans. We performed a retrospective analysis of the data of 14 patients (sex, nine men and five women; age, 37–70 years) who underwent medial opening-wedge high tibial osteotomy (MOWHTO) and were followed up for 12 months after surgery. Two wedge-shaped β-TCPs (one UDPTCP and one SPTCP) were cut and placed parallel to each other in the gap. In Group A (eight knees), UDPTCP was implanted anteriorly and SPTCP posteriorly, while in Group B (six knees), SPTCP was implanted anteriorly and UDPTCP posteriorly. Computed tomography (CT) was performed at 1 week, 6 months, and 12 months after surgery, with the CT attenuation values calculated for UDPTCP and SPTCP. In Groups A and B, the CT attenuation values for UDPTCP were significantly lower at 6 and 12 months after surgery compared to those at 1 week (P < 0.05); nevertheless, no statistical difference in the comparison with SPTCP was observed. After a short-term follow-up of 12 months following MOWHTO, UDPTCP provided earlier bone remodeling than SPTCP. This outcome was achieved regardless of the position, anterior or posterior, in the MOWHTO gap.

## Introduction

Autologous bone is the gold standard to fill bone defects. However, autologous bone harvesting has limited bone supply and is associated with potential complications, such as pain at the donor site and increased risk of infection^1^. To solve this problem, synthetic bone graft substitutes, such as hydroxyapatite (HAp)^2^ and β-tricalcium phosphate (TCP)^3^, have been developed. Although these materials have limited osteoinductivity^4^, β-TCP has been replaced by newly formed bone^5^ because of its osteoconductivity and is commonly used in clinical practice.

**Figure 1:**
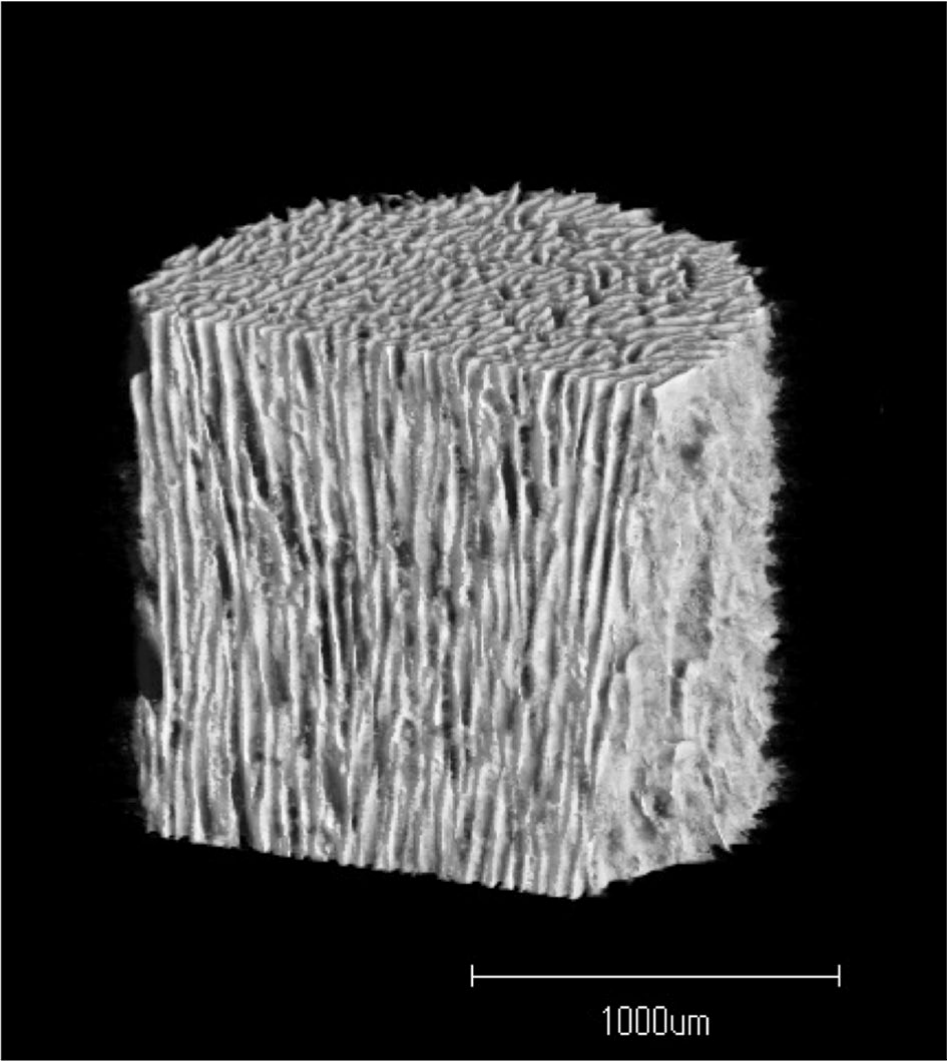
Three-dimensional micro-computed tomography image of unidirectional porous β-tricalcium phosphate (provided by Kuraray) showing unidirectional pores in the vertical direction and some interconnection toward the horizontal direction at the top.

Affinos (Kuraray, Tokyo, Japan) is a unidirectional porous β-TCP (UDPTCP), consisting of a novel porous artificial bone with a porosity of 57 ± 5%, in which communication holes 25–300 μm in diameter, are arranged in one direction (Fig. 1). These characteristics of UDPTCP allow for rapid penetration of the tissue into the material, whereas a good balance between bone formation and material resorption has been observed in a rabbit bone deficit model^6^. Medial opening-wedge high tibial osteotomy (MOWHTO) is a useful surgical option for the treatment of medial compartmental osteoarthritis and osteonecrosis of the knee^7,8^. However, MOWHTO has the disadvantage of creating a large vacant space in the proximal tibia^9^. Autologous bone and HAp have traditionally been used to fill this gap^10^. Recently, spherical porous β-TCP (SPTCP) with a porosity of 60% and interconnected pores (100–400 μm in diameter), which are not arranged in one direction, are increasingly being used, and a previous study reported that it had excellent stability and bone remodeling capacity^11^.

To fill the MOWHTO gap, two spacers can be implanted parallel to each other, allowing the possibility of comparing the remodeling capacity of different artificial bone types within the same individual. In this study, we investigated the differences between the bone remodeling of UDPTCP and SPTCP, radiologically in humans. On the basis of the characteristics of each material, we hypothesized that bone remodeling would be observed earlier with UDPTCP than with SPTCP. This study was approved and informed consent was waived by the Medical Ethical Committee of University of Tsukuba (no. R02-34) because of the retrospective design of the study. This study was conducted in compliance with relevant guidelines.

## Results

**Figure 2:**
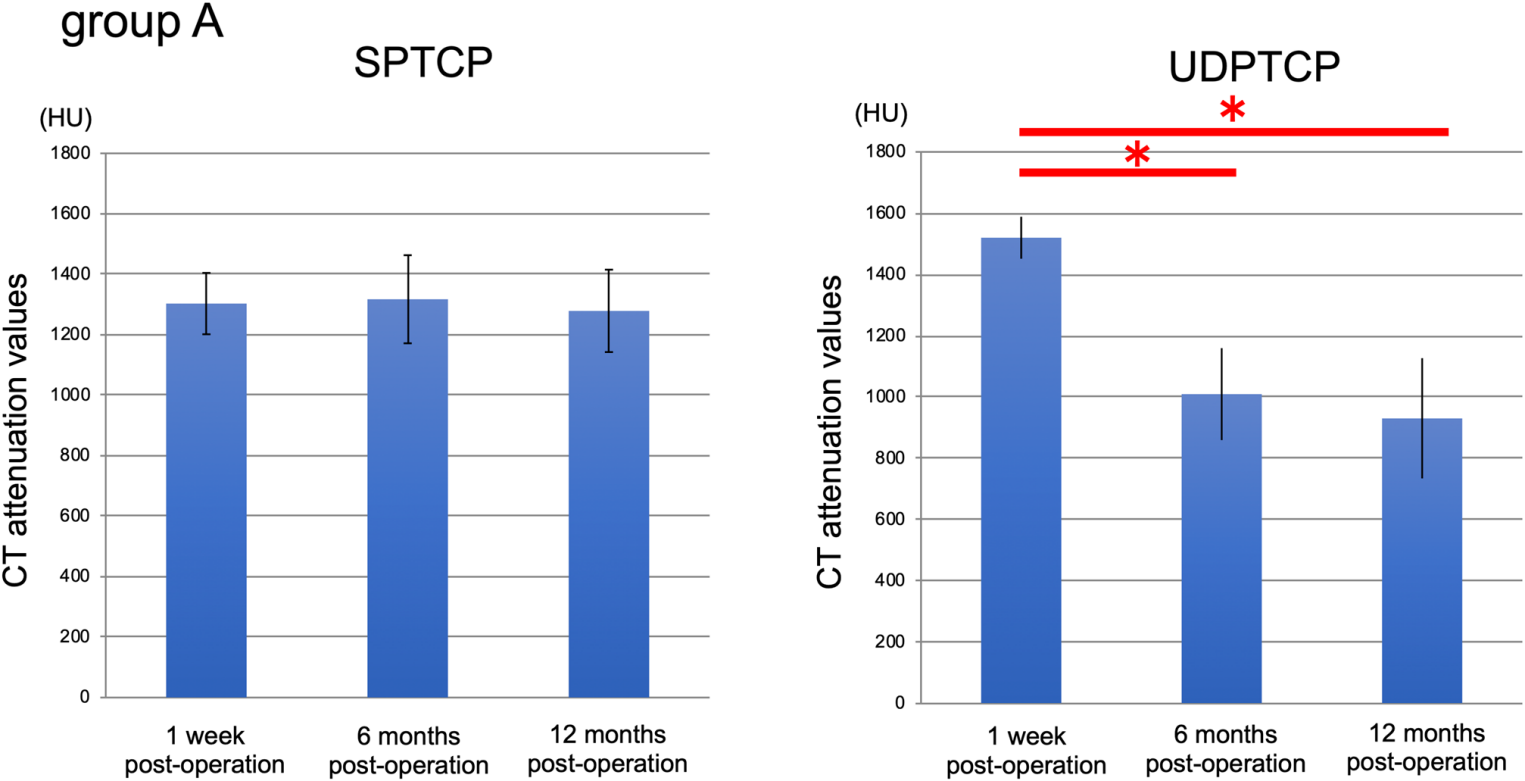
CT attenuation values (HU) for UDPTCP and spherical porous β-tricalcium phosphate at 1 week, 6 months, and 12 months after surgery in Group A. For UDPTCP, there was a significant attenuation between 1 week and 6 months, and between 1 week and 12 months (P < 0.05), with no statistical difference in the CT attenuation values for SPTCP. *CT* computed tomography, *HU* Hounsfield units, *SPTCP* spherical porous β-tricalcium phosphate, *UDPTCP* unidirectional porous β-tricalcium phosphate.

**Figure 3:**
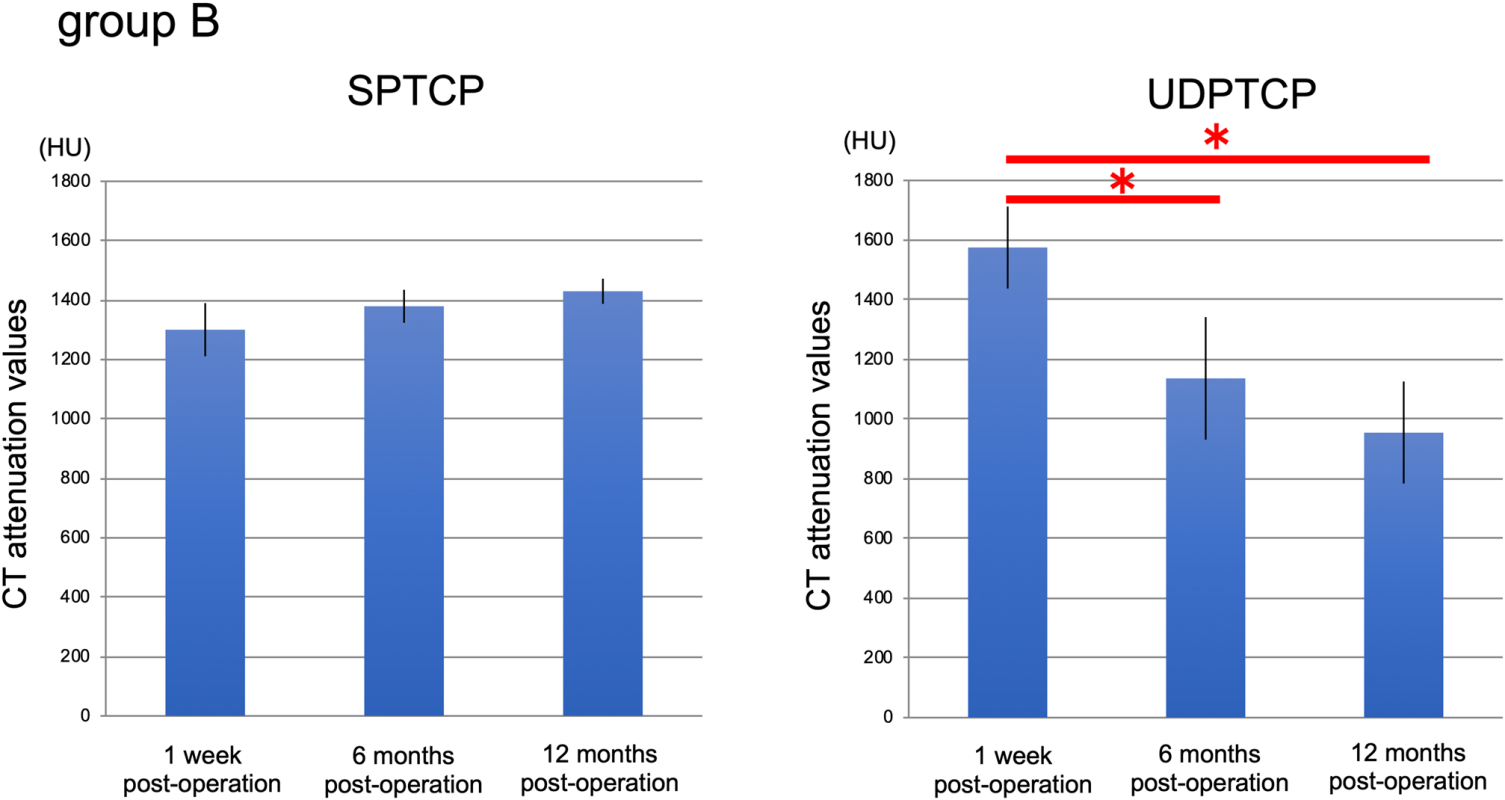
CT attenuation values (HU) for UDPTCP and spherical porous β-tricalcium phosphate at 1 week, 6 months, and 12 months after surgery in Group B. For UDPTCP, there was a significant attenuation between 1 week and 6 months, and between 1 week and 12 months (P < 0.05), with no statistical difference in the CT attenuation values for SPTCP. *CT* computed tomography, *HU* Hounsfield units, *SPTCP* spherical porous β-tricalcium phosphate, *UDPTCP* unidirectional porous β-tricalcium phosphate.

The mean ages of Groups A and B were 57.8 (range, 50–70) and 57.0 (range, 37–64) years, respectively. Relevant demographics for the two groups [sex, body mass index, pre- and post-operative femorotibial angle (FTA), and the mean opening-gap after osteotomy] are reported in Table 1, with no between-group differences noted. The time-dependent change in computed tomography (CT) attenuation values in Groups A and B at 1 week, 6 months, and 12 months after surgery were as follows: Group A [SPTCP, 1303 ± 101, 1317 ± 145, and 1279 ± 136 Hounsfield units (HU), respectively; UDPTCP, 1575 ± 137, 1136 ± 205, and 955 ± 170 HU, respectively]; Group B (SPTCP, 1300 ± 89, 1379 ± 55, and 1329 ± 42 HU, respectively; UDPTCP, 1520 ± 68, 1009 ± 149, and 930 ± 196 HU, respectively). For UDPTCP, the CT attenuation values were significantly lower at 6 and 12 months than at 1-week post-surgery (P < 0.05); however, there was no statistical difference in the time-dependent CT attenuation values for SPTCP (Figs. 2, 3). The comparison of the CT attenuation values between the two artificial bones at each time-point were as follows. In Group A, the CT attenuation value was significantly higher in UDPTCP compared to that in SPTCP (P < 0.05) at 1-week post-surgery, with no difference at 6 months. Moreover, the CT attenuation value was significantly lower in UDPTCP compared to that in SPTCP at 12 months (P < 0.05) was observed. In Group B, the CT attenuation value was significantly higher in UDPTCP compared to that in SPTCP (P < 0.05) at 1-week post-surgery. Moreover, the CT attenuation value was significantly lower in UDPTCP compared to that in SPTCP at 6 and 12 months (P < 0.05).

**Table 1:** Background characteristics of patients.

	Group A	Group B	P value
Age (years)	57.8 (6.9)	57.0 (11.0)	n.s.
Male/female (patients)	6/2	3/3	n.s.
Body mass index (kg/m^2^)	25.5 (3.0)	28.5 (3.5)	n.s.
Preoperative FTA (degree)	181.2 (1.2)	180.6 (2.2)	n.s.
Postoperative FTA (degree)	171.7 (0.8)	172.1 (3.3)	n.s.
Opening gap (mm)	13.3 (1.5)	13.0 (2.6)	n.s.

Mean (standard deviation).

*n.s.* non-significant, *SPTCP* spherical porous β-tricalcium phosphate, *TCP* tricalcium phosphate, *UDPTCP* unidirectional porous β-tricalcium phosphate.

## Discussion

This study showed that CT attenuation values decreased earlier for UDPTCP than for SPTCP when these two materials were used as a bone substitute for MOWHTO in the same individual. In previous reports, the CT attenuation value of the proximal tibia after OWHTO was 100–200 HU^12,13^, and the fact that the UDPTCP reached that value earlier compared to SPTCP indicated that UDPTCP promoted faster bone remodeling.

The strength of our study was that the two types of artificial bone were compared in the same individual and examined at different locations, namely an anterior or posterior position in the MOWHTO gap. Therefore, we assessed the bone remodeling potential of UDPTCP and compared it with that of SPTCP in the same environment. Considering that the sagittal mechanical axis of the lower extremity is not located at the center of the proximal tibia^14^, the loading conditions on the two artificial bone spacers could differ depending on whether they are placed anteriorly or posteriorly in the MOWHTO gap. In our study, we completed an in vivo assessment of the two artificial bone types for the anterior and posterior positions in the MOWHTO gap. Regardless of the location and, thus, loading, the CT attenuation values decreased faster for UDPTCP than for SPTCP.

UDPTCP and SPTCP have a different pore structure but have almost the same porosity (57% and 60%, respectively). UDPTCP is characterized by an interconnected network of pores that are aligned in a single direction. Makihara et al.^6^ demonstrated that this characteristic of UDPTCP allowed the rapid penetration of the tissue into the material when implanted in the tibia of rabbits. Similarly, using a rat model, Murayama et al.^15^ reported a faster infiltration of the bone into UDPTCP than into other types of β-TCP, resulting in a more rapid formation of capillaries throughout the UDPTCP. To the best of our knowledge, this was the first study that evaluated β-TCP remodeling in different structures and compared the CT attenuation values in humans. Interestingly, our findings were consistent with those of animal studies. However, we should consider that a simple comparison could not be made, as a previous report has suggested that bone remodeling in humans and animals was different^16^.

Although no clinical trial has directly compared SPTCP and UDPTCP, the benefits of UDPTCP in practice have been reported in a few studies. For calcaneal fractures, UDPTCP was replaced with autogenous bone at 6 months post-surgery, with favorable clinical outcomes obtained^17^. UDPTCP also provided good clinical potential as a bone substitute to fill gaps after the treatment of benign bone tumors of the hand and vertebral fractures^18^. Additionally, in an image-based assessment of lateral interbody fusion in the lumbar spine, UDPTCP was not inferior to the autologous bone graft^19^. In this study, we focused only on bone remodeling, but clinical outcomes should also be evaluated in the future.

However, the limitations of our study should be acknowledged. First, the sample size of the study was small. Second, our last assessment was performed at 12 months post-surgery; thus, longer-term differences between SPTCP and UDPTCP on bone remodeling were not considered. Third, we measured the CT attenuation values at only one point (i.e., at the center of the osteotomy). A previous work indicated that bone remodeling began from the TCP and the bone contact area in OWHTO^20^; therefore, the CT attenuation values may have been measured at the last area to be remodeled, and decrease in the CT attenuation values at this area implied that other areas had also been remodeled. Fourth, in this study, SPTCP and UDPTCP were implanted in the same gap, which makes it impossible to avoid their mutual influence. Finally, we did not perform any histological assessment of the bone union site. Therefore, further research is needed to clarify the remodeling process in detail.

In conclusion, over a short-term follow-up of 12 months after MOWHTO, UDPTCP provided earlier bone remodeling than SPTCP, as quantified by the CT attenuation findings. This outcome was achieved regardless of the position, anterior or posterior, in the MOWHTO gap.

## Methods

### Study group

This was a retrospective comparative study of 14 patients who underwent MOWHTO, using a locking compression plate. Of these, eight patients were enrolled between January and December 2017, in whom UDPTCP (Affinos) and SPTCP (Osferion 60, Olympus Terumo Biomaterials, Tokyo, Japan) were implanted in the anterior and the posterior spacer (Group A), respectively. The other six patients were enrolled between January 2018 and March 2019; in these patients, SPTCP and UDPTCP were implanted anteriorly and posteriorly, respectively (Group B).

### Description of the surgery

**Figure 4:**
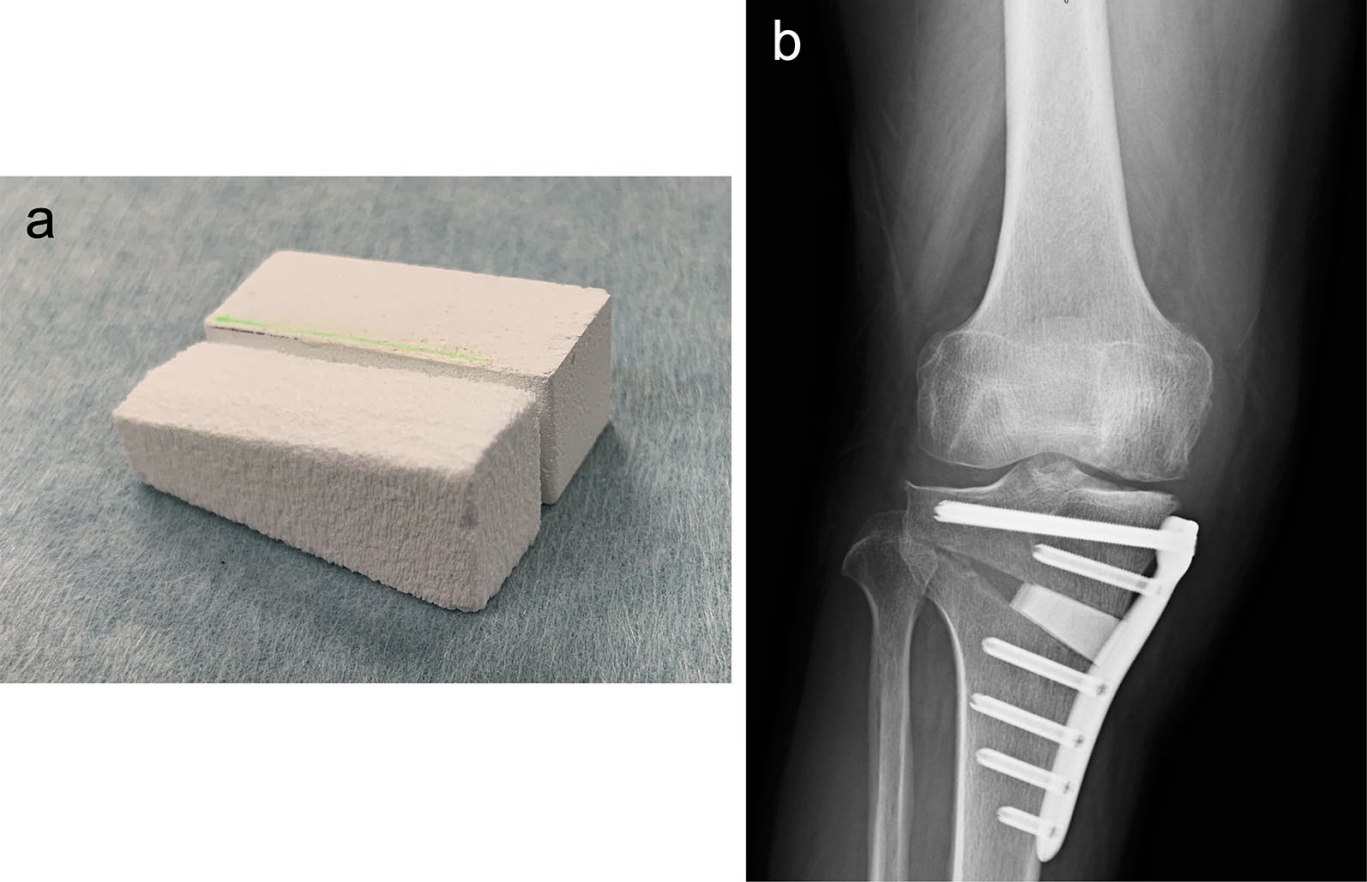
SPTCP and UDPTCP wedges were formed to the same size of the opening gap (**a**) and implanted parallel into the anterior and posterior parts of the osteotomy site (**b**). *SPTCP* spherical porous β-tricalcium phosphate, *UDPTCP* unidirectional porous β-tricalcium phosphate.

The surgery was performed by two surgeons using the standard MOWHTO protocol, after accurate pre-operative planning of the osteotomy on standing full-limb anterior–posterior (AP) radiographs^21^. A biplanar osteotomy was performed with a horizontal cut of the posterior two-third of the proximal tibia and a 100° angulated cut ascending anteriorly. The osteotomy site was opened and the gap created was filled with bone substitutes. From a rectangular block of SPTCP or UDPTCP, we created two wedge-shaped spacers, implanted parallel into the anterior and posterior parts of the opening gap (Fig. 4). In Group A, UDPTCP and SPTCP were used in the anterior and the posterior spacer, respectively. In contrast, in Group B, SPTCP and UDPTCP were used in the anterior and the posterior spacer, respectively.

The biomechanical characteristics of the SPTCP and UDPTCP are shown in Table 2. UDPTCP was implanted with the pores within the material oriented parallel to the tibial axis. A locking compression plate (TriS Medial HTO Plate System, Olympus Terumo Biomaterials, Tokyo, Japan) was used to fix the MOWHTO site. The patients were non-weight-bearing for 1 week after surgery, followed by partial weight-bearing, with full-body weight-bearing permitted at 4 weeks after surgery.

**Table 2:** Biomaterial characteristics of artificial bone.

	SPTCP	UDPTCP
Material	β-TCP	β-TCP
Structure	Spherical porous structure	Unidirectional porous structure
Porosity (%)	60	57
Pore diameter (μm)	100–400	25–300

*SPTCP* spherical porous β-tricalcium phosphate, *TCP* tricalcium phosphate, *UDPTCP* unidirectional porous β-tricalcium phosphate.

### Assessment of bone remodeling

**Figure 5:**
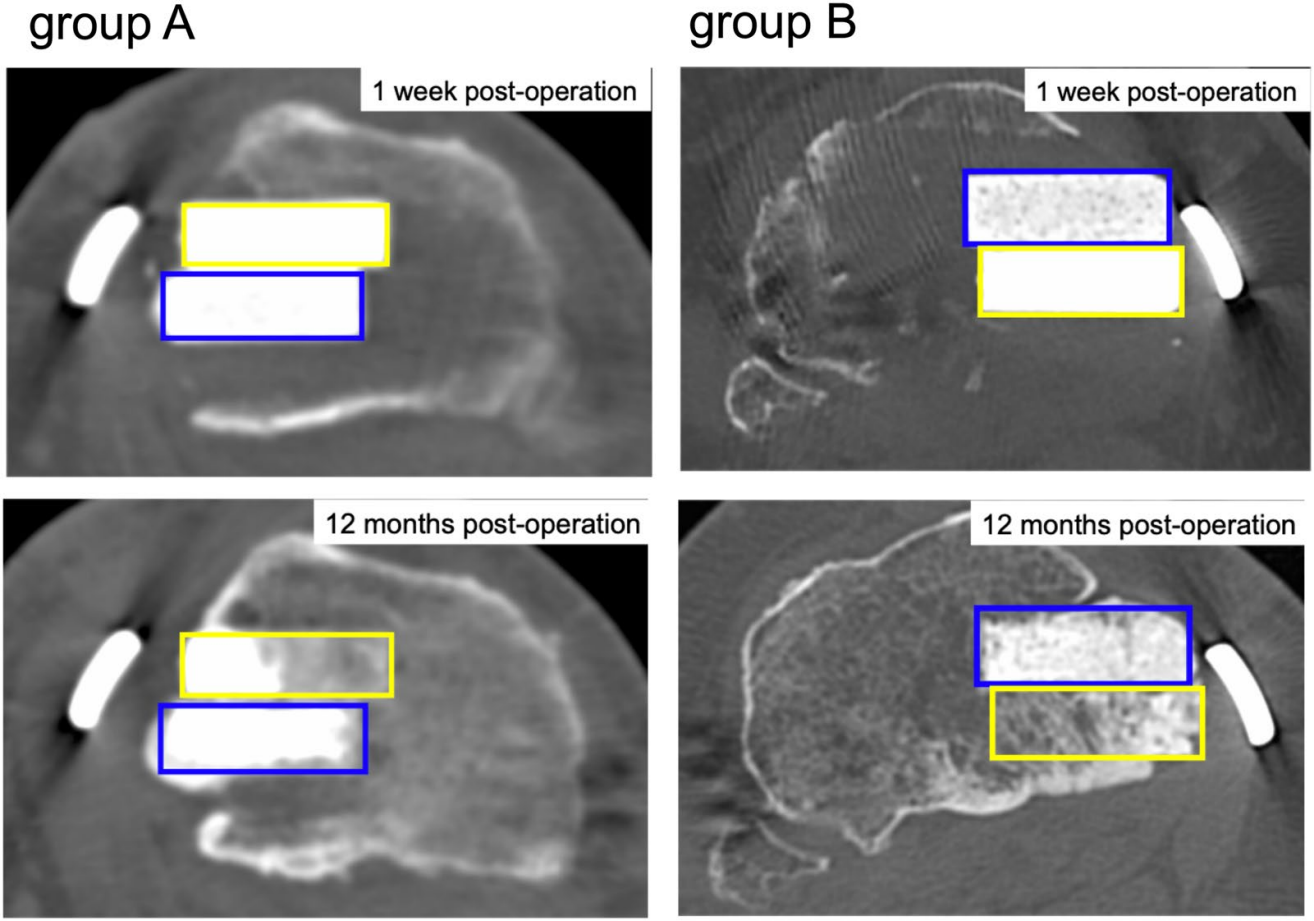
CT images showing the center of the osteotomy plane for Groups A and B. The mean CT value (HU) of the area implanted with UDPTCP (yellow rectangle) and the area implanted with SPTCP (blue rectangle). *CT* computed tomography, *HU* Hounsfield units, *SPTCP* spherical porous β-tricalcium phosphate, *UDPTCP* unidirectional porous β-tricalcium phosphate.

CT evaluations were performed at 1 week, 6 months, and 12 months post-operatively, to assess bone remodeling. The method reported by Tanaka et al.^12^ was used. Especially, CT images obtained parallel to the osteotomy plane and bone remodeling were assessed from images obtained at the center of the osteotomy plane. The CT image data were divided into two areas (UDPTCP and SPTCP) and the CT attenuation values (in HU) of each area were analyzed (Fig. 5) using the imaging software Osirix (Pixmeo, Geneva, Switzerland).

### Statistical analysis

The CT attenuation values were reported as mean ± standard deviations. One-way ANOVA with the Tukey–Kramer post-hoc test were used to examine the time-dependent change in the CT attenuation values for UDPTCP and for SPTCP. The Mann–Whitney *U* test was used to compare the CT attenuation values between UDPTCP and SPTCP at each measurement time-point. The level of statistical significance was set at P < 0.05. All statistical analyses were performed using SPSS Statistics software, version 26.0 (IBM Corp., Armonk, NY, USA).

### Ethics approval

This study was approved by the Medical Ethical Committee of University of Tsukuba (No. R02-34).

### Consent to participate and publication

Written informed consent was waived by the Medical Ethical Committee of University of Tsukuba (No. R02-34) because of the retrospective design, but instead this study’s protocol is open to the public.

## Data Availability

All data generated or analyzed during this study are included in this published article.

## References

[1] Ebraheim NA, Elgafy H, Xu R (2001). Bone-graft harvesting from iliac and fibular donor sites: techniques and complications. J. Am. Acad. Orthop. Surg..

[2] Uchida A (1990). The use of calcium hydroxyapatite ceramic in bone tumour surgery. J. Bone Joint Surg. Br..

[3] Kondo N (2005). Bone formation and resorption of highly purified β-tricalcium phosphate in the rat femoral condyle. Biomaterials.

[4] Salgado AJ, Coutinho OP, Reis RL (2004). Bone tissue engineering: state of the art and future trends. Macromol. Biosci..

[5] Ogose A (2005). Comparison of hydroxyapatite and beta tricalcium phosphate as bone substitutes after excision of bone tumors. J. Biomed. Mater. Res. B Appl. Biomater..

[6] Makihara T, Sakane M, Noguchi H, Yamazaki M (2016). The balance between bone formation and material resorption in unidirectional porous β-Tricalcium phosphate implanted in a rabbit tibia. Key Eng. Mater..

[7] Coventry MB, Ilstrup DM, Wallrichs SL (1993). Proximal tibial osteotomy. A critical long-term study of eighty-seven cases. J. Bone Joint Surg. Am..

[8] Marti CB, Rodriguez M, Zanetti M, Romero J (2000). Spontaneous osteonecrosis of the medial compartment of the knee: a MRI follow-up after conservative and operative treatment, preliminary results. Knee Surg. Sports Traumatol. Arthrosc..

[9] Gouin F (2010). Open wedge high tibial osteotomies: calcium-phosphate ceramic spacer versus autologous bonegraft. Orthop. Traumatol. Surg. Res..

[10] Koshino T, Murase T, Saito T (2003). Medial opening-wedge high tibial osteotomy with use of porous hydroxyapatite to treat medial compartment osteoarthritis of the knee. J. Bone Joint Surg. Am..

[11] Takeuchi R (2008). Simultaneous bilateral opening-wedge high tibial osteotomy with early full weight-bearing exercise. Knee Surg. Sports Traumatol. Arthrosc..

[12] Tanaka T (2015). A novel evaluation system to monitor bone formation and β-tricalcium phosphate resorption in opening wedge high tibial osteotomy. Knee Surg. Sports Traumatol. Arthrosc..

[13] Conteduca F (2016). Nanohydroxyapatite promotes the healing process in open-wedge high tibial osteotomy: a CT study. Knee.

[14] Minoda Y (2008). Sagittal alignment of the lower extremity while standing in Japanese male. Arch. Orthop. Trauma Surg..

[15] Murayama A, Ajiki T, Hayashi Y, Takeshita K (2019). A unidirectional porous beta-tricalcium phosphate promotes angiogenesis in a vascularized pedicle rat model. J. Orthop. Sci..

[16] Wancket LM (2015). Animal models for evaluation of bone implants and devices: comparative bone structure and common model uses. Vet. Pathol..

[17] Izawa S (2017). The use of unidirectional porous β-tricarcium phosphate in surgery for calcaneal fractures: a report of four cases. Foot Ankle Online J..

[18] Ikumi A, Funayama T, Tsukanishi T, Noguchi H, Yamazaki M (2018). Novel unidirectional porous β-tricalcium phosphate used as a bone substitute after excision of benign bone tumors of the hand: a case series. J. Hand Surg. Asian Pac..

[19] Kumagai H (2019). Unidirectional porous β-tricalcium phosphate induces bony fusion in lateral lumbar interbody fusion. J. Clin. Neurosci..

[20] Van Hemert WLW, Willems K, Anderson PG, Van Heerwaarden RJ, Wymenga AB (2004). Tricalcium phosphate granules or rigid wedge preforms in open wedge high tibial osteotomy: a radiological study with a new evaluation system. Knee.

[21] Pape D, Rupp S (2007). Preoperative planning for high tibial osteotomies. Oper. Tech. Orthop..

